# Resistance-Related l-Pyroglutamic Acid Affects the Biosynthesis of Trichothecenes and Phenylpropanoids by *F. graminearum* Sensu Stricto

**DOI:** 10.3390/toxins10120492

**Published:** 2018-11-24

**Authors:** Katarzyna Bilska, Kinga Stuper-Szablewska, Tomasz Kulik, Maciej Buśko, Dariusz Załuski, Juliusz Perkowski

**Affiliations:** 1 Department of Botany and Nature Protection, University of Warmia and Mazury in Olsztyn Plac Łódzki 1, 10-727 Olsztyn, Poland; tomaszkulik76@gmail.com; 2 Department of Chemistry, Poznan University of Life Sciences, Wojska Polskiego 75, 60-637 Poznan, Poland; kstuper@up.poznan.pl (K.S.-S.); mabu@up.poznan.pl (M.B.); julperk@up.poznan.pl (J.P.); 3 Department of Plant Breeding and Seed Production, University of Warmia and Mazury in Olsztyn, Plac Łódzki 3, 10-727 Olsztyn, Poland; dariusz.zaluski@uwm.edu.pl

**Keywords:** *Fusarium*, l-pyroglutamic acid, trichothecenes, phenolic acids, flavonoids, gene expression

## Abstract

Fungicide application remains amongst the most widely used methods of fungal control in agroecosystems. However, the extensive use of fungicides poses hazards to human health and the natural environment and does not always ensure the effective decrease of mycotoxins in food and feed. Nowadays, the rising threat from mycotoxin contamination of staple foods has stimulated efforts in developing alternative strategies to control plant pathogenic fungi. A substantial effort is focused on the identification of plant-derived compounds inhibiting mycotoxin production by plant pathogenic fungi. l-Pyroglutamic acid has recently been suggested as playing a role in the response of barley to toxigenic Fusaria. Considering the above, we studied the response of various strains of *F. graminearum* sensu stricto to different levels of l-pyroglutamic acid on solid YES (yeast extract sucrose) media. l-Pyroglutamic acid decreased the accumulation of trichothecenes in all examined strains. Gene expression studies addressing *Tri* genes (*Tri*4, *Tri*5, and *Tri*10), which induce the biosynthesis of trichothecenes, revealed the production of mycotoxins by l-pyroglutamic acid to be inhibited at the transcriptional level. Besides inhibitory effects on mycotoxin production, l-pyroglutamic acid exhibited variable and concentration-related effects on phenylpropanoid production by fungi. Accumulation of most of the fungal-derived phenolic acids decreased in the presence of 100 and 400 µg/g of l-pyroglutamic acid. However, a higher dose (800 µg/g) of l-pyroglutamic acid increased the accumulation of *trans*-cinnamic acid in the media. The accumulation of fungal-derived naringenin increased in the presence of l-pyroglutamic acid. Contrasting results were obtained for quercetin, apigenin, luteolin, and kaempferol, the accumulation of which decreased in the samples treated with 100 and 400 µg/g of l-pyroglutamic acid, whereas the highest l-pyroglutamic acid concentration (800 µg/g) seemed to induce their biosynthesis. The results obtained in this study provide new insights for breeders involved in studies on resistance against Fusaria.

## 1. Introduction

A common ascomycete fungus, *Fusarium graminearum* sensu stricto (s.s.) remains a severe cereal pathogen worldwide. On small-grain cereals such as barley and wheat, this necrotroph can cause two different diseases: FHB (*Fusarium* head blight, referred to also as ear blight or scab) and FRR (*Fusarium* foot and root rot) [1,2]. FHB causes serious yield losses and decline in seed quality via grain contamination with type B trichothecenes [3].

Type B trichothecenes inhibit eukaryotic protein synthesis by binding to the 60S ribosomal unit and by interacting with the peptidyl transferase enzyme [4]. Consumption of contaminated food may cause food poisoning manifested by nausea, vomiting, and diarrhea, as well as headache, dizziness, and fever [5,6]. Higher doses of trichothecenes may damage bone marrow, the thymus, and the lymphoid and epithelial cells of the gastrointestinal mucosa [7]. Trichothecenes are heat-stable and therefore are not fully degraded by the cooking of food or by the processes used in food manufacturing [8,9].

The use of fungicides, principally azoles, is a major method for FHB management [10]. The control of Fusaria by fungicides often proves ineffective, mainly because of difficulties in timing and administration of the fungicides to the ear in the critical infection period during anthesis [11]. Furthermore, *F. graminearum* s.s. can develop increased resistance to this class of fungicides [12,13,14,15,16], while residues of massively used azoles can pervade into and persist in the environment, thereby posing high risk to nontarget organisms [17].

In recent years, accelerated efforts have been undertaken into the development of environmentally friendly disease management approaches [18]. Breeding of cultivars exhibiting high resistance against Fusaria remains the most promising strategy. Thus, in planta studies have been conducted to identify metabolites associated with resistance to fungal pathogens [19,20,21,22,23,24]. Other, simultaneous efforts have included in vitro studies evaluating the effect of these metabolites on fungal secondary metabolism. To date, phenylpropanoids have attracted the greatest attention in both in planta and in vitro studies [2,3,25,26,27,28,29,30]. The latter ones have shown that phenylpropanoids display molecule-dependent inhibitory effects on mycotoxin production by fungi [2,3,27], including *F. graminearum* s.s. [28,29,30]. 

In this study, we characterized the impact of l-pyroglutamic acid on the secondary metabolism of *F. graminearum* s.s. This organic acid was previously identified as a resistance-related (RR) metabolite in barley in response to *Fusarium* infection [21,22,31]. l-Pyroglutamic acid is an analogue and a potential precursor of glutamate [32], which is essential for the biosynthesis of glutathione. The latter is known as a powerful antioxidant [33]. Previous studies have shown that l-pyroglutamic acid exhibits both antibacterial [34] and antifungal activity against phytopathogenic *Pseudoperonospora cubensis* and *Phytophthora infestans* [35]. To the best of our knowledge, there are no reports characterizing the impact of this compound on Fusaria.

We studied the effect of l-pyroglutamic acid on the accumulation of trichothecenes in the culture media as well as on the expression of the *Tri* genes (*Tri*4, *Tri*5, and *Tri*10) which induce their biosynthesis. We found that both gene expression and trichothecene accumulation were inhibited in all studied strains treated with l-pyroglutamic acid. In the present study, we also showed that the treatment with l-pyroglutamic acid impacts fungal phenolic acid and flavonoid profiles. The results obtained in this study provide new insights, especially for breeders involved in studies on resistance against Fusaria.

## 2. Results and Discussion

### 2.1. Exogenous l-Pyroglutamic Acid Affects the Production of Phenolic Acids and Flavonoids by Fungal Strains

After 21 days of incubation, fungi that were exposed to 100, 400, or 800 µg/g of l-pyroglutamic acid reduced its concentration by 90–91%, 46–51%, and 56–60%, respectively. The substantial reduction of exogenous l-pyroglutamic acid might be linked to its efficient metabolization by fungi. l-pyroglutamic acid can be easily converted to glutamate by 5-oxoprolinase [36,37] and has also been found at the N-terminus of many peptides and proteins [32]. 

Fungal strains used in this study produced phenolic acids and phenylpropanoids on the solid YES (yeast extract sucrose) medium (Supplementary Table S1), which is in agreement with findings from our previous studies [28,29,30]. After 21 days of incubation with l-pyroglutamic acid, changes were observed in fungal secondary metabolite profiles. The total phenolic acids content decreased by 3.5- to 5-fold (depending on l-pyroglutamic acid concentration) in the fungal cultures exposed to l-pyroglutamic acid. It is worth noting, however, that in the case of two phenolic acids (protocatechuic and *trans*-cinnamic acids), exceptional results were obtained. No significant changes in protocatechuic acid were found between the samples treated with l-pyroglutamic acid and controls (YES + fungal controls). The second exception concerned the higher concentration of *trans-*cinnamic acid after incubation with 800 µg/g of l-pyroglutamic acid. Nonoxidative deamination of l-phenylalanine to *trans*-cinnamic acid by phenylalanine ammonia-lyase (PAL; E.C 4.3.1.5) is a key step of phenylpropanoid biosynthesis. Accumulation of *trans*-cinnamic acid was also identified by Kulik et al. [29] after *Fusarium* strains’ treatment with chlorogenic acid and by Bilska et al. [30] after their treatment with luteolin, kaempferol, and quercetin. The higher concentration of *trans*-cinnamic acid may indicate that the activity of PAL responsible for phenylpropanoid production was not completely inhibited by l-pyroglutamic acid. Increased accumulation of *trans*-cinnamic acid may also suggest that l-pyroglutamic acid has an inhibitory effect on enzymes converting this first intermediate of all phenylpropanoids further. 

**Figure 1:**
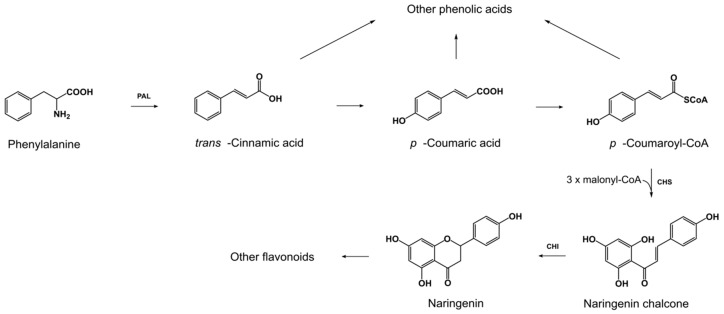
Scheme of the biosynthesis of selected phenylpropanoids.

In this study, all three concentrations of l-pyroglutamic acid induced the biosynthesis of naringenin. Accumulation of other flavonoids (quercetin, apigenin, luteolin, and kaempferol) was inhibited in the samples treated with two lower concentrations of l-pyroglutamic acid (100 and 400 µg/g), whereas the highest acid concentration (800 µg/g) seemed to induce their biosynthesis. The sum of flavonoids increased almost 2.5-fold after incubation with 800 µg/g of l-pyroglutamic acid. It is unclear why the exposure to lower amounts of l-pyroglutamic acid suppressed the production of some flavonoids by fungal strains. However, the increased concentration of naringenin and other flavonoids may be indicative of *trans*-cinnamic acid conversion to naringenin chalcone, which is a precursor of naringenin (Figure 1). It seems that l-pyroglutamic acid stimulates the activity of enzymes responsible for naringenin biosynthesis: chalcone synthase (CHS) and chalcone isomerase (CHI) (Figure 1), whereas the activity of enzymes catalyzing the conversion of phenolic acids could be inhibited. 

### 2.2. Effect of l-Pyroglutamic Acid on the Accumulation of Trichothecenes in Culture Media

The effect of l-pyroglutamic acid on the production of trichothecenes by fungal strains was established based on trichothecene counts determined on the treated and nontreated plates (YES + fungal controls). l-Pyroglutamic acid had strong inhibitory effects on trichothecene accumulation in the case of all three studied *F. graminearum* s.s. strains; however, the degree of toxin inhibition was strain-dependent (Table 1). *F. graminearum* s.s. strains kept at 100, 400, or 800 µg/g of l-pyroglutamic acid reduced the trichothecene concentration by 83–89%, 56–93%, and 67–97%, respectively.

Previous studies linked the inhibitory effect of phenylpropanoids to their antioxidant and antiradical properties [29,30,38]. The stronger the antioxidant/antiradical property of the phenylpropanoid, the more efficient the inhibitory effect on mycotoxin production. Indeed, as shown in Table 2, l-pyroglutamic acid displays high antioxidant activity compared to other phenolic acids. It exhibits stronger antioxidant properties than chlorogenic and sinapic acids, which as shown previously, displayed inhibitory effects on mycotoxin production by Fusaria. The strong inhibitory effects of l-pyroglutamic acid may also be linked to its high radical-scavenging activity, which appears to be higher than that of *tran*s-cinnamic acid (Table 2), exhibiting strong inhibitory effects on trichothecene biosynthesis by *Fusarium* spp. [29].

### 2.3. Effect of l-Pyroglutamic Acid on the Expression of Tri Genes

The effect of l-pyroglutamic acid on *Tri* gene (*Tr*i4, *Tri*5, and *Tri*10) expression was evaluated on day three of incubation (Table 1). Gene expression studies showed the production of mycotoxins by l-pyroglutamic acid to be inhibited at the transcriptional level. l-Pyroglutamic acid inhibited the activity of *Tri* genes; however, this inhibition showed no significant fold-change values (P(H1) = 0.001) in most samples treated with 100 µg/g of l-pyroglutamic acid. Expression of *Tri* genes is regulated by two genes: *Tri*10 and *Tri*6 [39,40,41]. *Tri*10 positively regulates the transcription of *Tri*6 [41], which affects the expression of other *Tri* genes [39,40]. In our study, *Tri*10 transcription generally correlated with *Tri*4 and *Tri*5 expression; however, there were some exceptions. We noticed inhibition of *Tri*4 and *Tri*5 gene expression in MUCL 53455 strains in the presence of l-pyroglutamic acid, despite no changes observed in *Tri*10 expression. 

## 3. Conclusions

l-Pyroglutamic acid decreased the accumulation of trichothecenes in all examined strains. Gene expression studies addressing *Tri* genes which induce the biosynthesis of trichothecenes (*Tri*4, *Tri*5, and *Tri*10) revealed the production of mycotoxins by l-pyroglutamic acid to be inhibited at the transcriptional level. Besides inhibitory effects on mycotoxin production, l-pyroglutamic acid exhibited variable and concentration-related effects on phenylpropanoid production by fungi.

## 4. Materials and Methods

### 4.1. Fungal Strains

*Fusarium graminearum* s.s. strains used in this study are summarized in Table 3. Detailed characteristics are provided in the ToxGen database [42]. All strains are maintained in the international fungal collections: Westerdijk Fungal Biodiversity Institute (Utrecht, the Netherlands), MUCL—MUCL Mycothèque de l’Université catholique de Louvain (Louvain-la-Neuve, Belgium), and ARS Culture Collection, USDA (Peoria, IL, US). 

### 4.2. Medium and Culture Conditions 

l-Pyroglutamic acid (dissolved in 10 mL of 96% ethanol) (Sigma–Aldrich, Saint Louis, MO, USA) was added to the YES (yeast extract sucrose) medium to obtain the final concentrations of: 100 μg/g, 400 μg/g, and 800 μg/g. The three acid concentrations corresponded to those used by Kulik et al. [28,29] and Bilska et al. [30]. Three different controls were used in this study: YES-only control (YES medium only), YES + l-pyroglutamic acid control (YES media supplemented with either 100, 400, or 800 μg/g of acid), and three YES + fungal controls (*F. graminearum* s.s. strains incubated on YES media). 

Petri dishes (Ø 80 mm) were inoculated from laboratory stock cultures (6–8 weeks old) and incubated at 25 °C (in triplicate) in the dark. Gene expression analyses were performed after 3 days of incubation, while chemical analyses (mycotoxins, flavonoids, and phenolic acids determination) were performed after 21 days of incubation. 

### 4.3. Determination of Contents of Phenolic Acids and Flavonoids in the Culture Medium 

Fungal-derived phenylpropanoid concentrations were established in dried fungal cultures after 21 days of incubation (Supplementary Table S1), as previously described by Kulik et al. [28,29] and Bilska et al. [30]. Alkaline and acid hydrolysis were conducted in sealed 17-mL culture tubes containing 0.2 g of fungal biomass. An ACQUITY H class UPLC (Ultra performance liquid chromatography) system equipped with a Waters ACQUITY Photodiode Array (PDA) detector (Waters, Milford, MA, USA) was used in the analysis. Concentrations of l-pyroglutamic acid were determined using an internal standard at a wavelength of λ = 310 nm. l-Pyroglutamic acid identification was based on a comparison of sample and standard retention times. A specific amount of the standard was added to the analyzed samples. In the next step, the analysis was repeated. The detection level was 1 μg/g. The retention time of l-pyroglutamic acid was 19.05 min.

### 4.4. Determination of the Antioxidant Capacity (VCEAC/L) and Radical Scavenging Activity (ABTS+) of l-Pyroglutamic Acid

VCEAC/L and ABTS+ assays of l-pyroglutamic acid were conducted according to the methods provided earlier by Kim et al. [43] and Re et al. [44], respectively. The results were expressed as ABTS (μmol TROLOX/100 g d.m.) and mg Vitamin C-equivalent antioxidant capacity per liter (mg VCEAC/L).

### 4.5. Analysis of Trichothecene Concentrations from Fungal Cultures 

GC-MS was used to determine levels of trichothecenes (DON- deoxynivalenol; NIV- nivalenol; 3-AcDON- 3-acetylodeoxynivalenol; 15-AcDON- 15-acetylodeoxynivalenol) in fungal cultures exposed or nonexposed (YES + fungal controls) to various concentrations of l-pyroglutamic acid, as described in a study by Perkowski et al. [45]. 

### 4.6. Extraction of Total RNA and Preparation of cDNA 

Mycelium from 3-day-old fungal cultures grown on the YES medium exposed or nonexposed (YES + fungal controls) to various concentrations of l-pyroglutamic acid was used for RNA extraction. Six biological replications were prepared for each studied condition. Total RNA was extracted using a Pure Link^®^ RNA MINI KIT (Life Technologies, Carlsbad, CA, USA) following the manufacturer’s recommendations. Extraction of RNA and reverse transcription were conducted according to Kulik et al. [46,47]. cDNA samples were stored at −25 °C.

### 4.7. Gene Expression Analysis 

In this study, expression of two genes (*Tri*4, *Tri*5) playing a fundamental role in trichothecene biosynthesis [48,49,50,51,52] and expression of the *Tri*10 regulatory gene [41] were analyzed. The elongation factor 1-alpha gene (*Ef1α*) was used as a reference gene. The experiment was conducted in accordance with Kulik et al. [28,29,46,47]. The REST 2009 software [53] was used to normalize the Ct (treshold cycle) values of the target (*Tri*4, *Tri*5, and *Tri*10) genes in control and treated samples, relative to the Ct values obtained for the reference *Ef1α* gene. 

### 4.8. Statistical Analyses

Statistical analyses were performed using STATISTICA (Data Analysis Software System, ver. 12.5; StatSoft Inc., Tulsa, OK, USA, 2014). Tukey’s HSD (honest significant difference) test (*p* < 0.05) was used to show the differences among mycotoxin levels. The production of phenylpropanoids (phenolic acids and flavonoids) was estimated by an analysis of the differences among phenylpropanoid levels between YES-only and YES + fungal controls using the Student’s *t*-test at *p* < 0.05. This statistical method was also used to test the impact of exogenously applied l-pyroglutamic acid on the accumulation of phenylpropanoids and to analyze the reduction of exogenous l-pyroglutamic acid. 

## Figures and Tables

**Table 1:** Trichothecene levels and relative quantification (RQ) of *Tri* transcripts of fungal strains incubated with different levels of l-pyroglutamic acid.

l-Pyroglutamic Acid Concentration	Strain	*Tri* Genotype	Trichothecene Levels (mg/kg) (*n* = 3 in Each Condition)					RQ (*n* = 6 in Each Condition)			Relative Radial Growth (*n* = 3 in Each Condition)
			DON	3-AcDON	15-AcDON	NIV	Total	*Tri*4	*Tri*5	*Tri*10	
**YES + Fungal Controls**	CBS 138561	15ADON	1.43 ± 0.7		1.47 ± 0.85		2.9				100
	CBS 119173	3ADON	49.55 ± 3.57	12.24 ± 0.76			61.79				100
	MUCL 53455	NIV				4.41 ± 0.18	4.41				100
**100 μg/g**	CBS 138561	15ADON	0.47 ± 0.02 (b)		0.03 ± 0.001 (b)		0.5	0.76 (0.635–0.905)	NS	NS	106.28 ± 5.02 (NS)
	CBS 119173	3ADON	5.05 ± 0.3 (d)	1.54 ± 0.03 (d)			6.59	0.349 (0.271–0.458)	NS	NS	95.63 ± 0.44 (NS)
	MUCL 53455	NIV				0.49 ± 0.04 (c)	0.49	NS	NS	NS	89.22 ± 0.43 (b)
**400 μg/g**	CBS 138561	15ADON	0.17 ± 0.007 (b)		0.02 ± 0.001 (b)		0.19	0.035 (0.028–0.044)	0.34 (0.272–0.429)	0.71 (0.615–0.83)	98.33 ±2.93 (NS)
	CBS 119173	3ADON	21.14 ± 0.85 (b)	5.95 ± 0.36 (b)			27.09	0.117 (0.088–0.148)	0.421 (0.28–0.637)	0.123 (0.098–0.1)	107.42 ± 2.62 (NS)
	MUCL 53455	NIV				0.6 ± 0.05 (bc)	0.6	0.558 (0.420–0.777)	0.645 (0.531–0.786)	NS	92.24 ± 2.59 (ab)
**800 μg/g**	CBS 138561	15ADON	0.08 ± 0.003 (b)		0.02 ± 0.001 (b)		0.1	0.065 (0.055–0.08)	0.25 (0.213–0.3)	0.427 (0.368–0.495)	94.14 ± 6.28 (NS)
	CBS 119173	3ADON	15.00 ± 0.3 (c)	4.4 ± 0.09 (c)			19.4	0.072 (0.003–0.456)	0.215 (0.143–0.325)	0.04 (0.008–0.097)	108.73 ± 11.79 (NS)
	MUCL 53455	NIV				0.79 ± 0.08 (b)	0.79	0.451 (0.362–0.563)	0.23 (0.161–0.342)	NS	82.76 ± 2.59 (b)
**Degree of mycotoxin inhibition:**		<25%	25–50%	50–75%	>75%						

(a), (b), (c), and (d) lettering indicates homogenous groups at *p* < 0.05 followed by the Tukey test; NS—not significant; DON- deoxynivalenol; NIV- nivalenol; 3-AcDON- 3-acetylodeoxynivalenol; 15-AcDON- 15-acetylodeoxynivalenol.

**Table 2:** Antioxidant capacity (VCEAC/L) and radical scavenging activity (ABTS) of l-pyroglutamic acid and selected phenolic acids.

Acid	VCEAC/L	ABTS (μmol TROLOX/100 g d.m.)
**l-Pyroglutamic Acid**	421.2	568.1
*tran*s-Cinnamic Acid *	812.3	314.9
Chlorogenic Acid *	12.2	57.1
Sinapic Acid **	121	194.5

* from Kulik et al. [29]; ** from Kulik et al. [28]; VCEAC- vitamin C equivalent antioxidant capacity; ABTS- 2,2’-azino-bis(3-ethylbenzothiazoline-6-sulphonic acid).

**Table 3:** List of *F. graminearum* s.s. isolates used in this study.

Species	Strain	Trichothecene Genotype	Origin, Host, and Year of Isolation
***F. graminearum* s.s.**	CBS 138561	15ADON	Poland, wheat, 2010
	CBS 119173, NRRL 38369	3ADON	USA, Louisiana, wheat, 2005
	MUCL 53455	NIV	Belgium, corn, 2007

## Supplementary Materials

The following are available online at http://www.mdpi.com/2072-6651/10/12/492/s1, Supplementary Table S1: Flavonoid and phenolic acid accumulation by *F. graminearum* s.s. strains after 21 d of incubation in the presence of l-pyroglutamic acid.
